# An analytic network process model to prioritize supply chain risks in green residential megaprojects

**DOI:** 10.1007/s12063-022-00288-2

**Published:** 2022-06-22

**Authors:** A. M. Alamdari, Y. Jabarzadeh, B. Adams, D. Samson, S. Khanmohammadi

**Affiliations:** 1 Department of Management, University of Tabriz, Tabriz, Iran; 2 Liverpool Business School, Liverpool John Moores University, Liverpool, UK; 3 Department of Mathematics, Florida State University, Tallahassee, USA; 4 Decision Lens Inc., 4075 Wilson Blvd Suite 700, Arlington, USA; 5 Department of Management and Marketing, The University of Melbourne, Melbourne, Australia; 6 Department of Electrical Engineering, University of Tabriz, Tabriz, Iran

**Keywords:** Supply chain risk, Green building megaprojects, Fuzzy analytic network process, Row sensitivity analysis

## Abstract

Megaprojects and specifically ‘green’ construction of residential megaprojects can contain significant risks of failure. To design proper risk mitigation strategies, after identifying key risk factors, the next step is to conduct assessments that would facilitate the process of risk element prioritization. Risk assessment comprises the establishment of factor interrelation and discerning the indicators of importance. This research proposes a novel version of an integrated prioritization method and analyzes twelve all-inclusive key supply chain oriented risk factors identified in a previous study. Through a comprehensive literature review three criteria, impact, probability, and manageability are selected. Also, a fourth criterion namely influence rate is included in the model, based on the driving powers that can also be derived from the Interpretive Structural Modeling’s (ISM) assessment. Fundamentally, the calculations hinge on the Analytic Network Process (ANP) method which provides an assessment of the alternatives’ weights based on pairwise comparisons concerning the criteria specified. To enhance the accuracy of the perceptive judgments of the expert panelists, a bell-shaped fuzzy function is used to convert the verbal statements to crisp values. In addition, Row Sensitivity Analysis is administered to check the stability of the results and provide predictive scenarios. To validate the model, a case study, located in Iran, was conducted, where an expert panel consisting of four individuals made the pair-wise comparisons through an ANP questionnaire. Results indicate priority and sensitivity of the alternatives concerning criteria, for the case under study.

## Introduction

The green construction industry aims to minimize resource depletion and emission of any kind of pollution, controlling energy consumption through environmentally friendly practices (Council and Council 2016). Contributing to sustainable development goals (Fei et al. 2021; Wen et al. 2020), green building projects have gained increasing attention over the past two decades for the significant role they can play in mitigating the impacts of climate change, improving the human health, and lowering the life-cycle cost (Wuni et al. 2019). Furthermore, operational measures such as improved efficiency or productivity can be achieved (Maditati et al. 2018), while large-scale green construction projects’ various stakeholders can also benefit from the institutionalization of green practices (Mok et al. 2018). In this regard, green supply chain management (GSCM) practices can result in improved business competitiveness (de Oliveira et al. 2018). Nevertheless, megaprojects, in particular, may still encounter operational problems during the project lifecycle, bringing them to a halt (Flyvbjerg 2014).

Construction megaprojects’ main challenge is to deal with the induced risks of failure due to ambiguity, uncertainty, and the dynamic nature of such complex projects (Lehtiranta 2011; Qazi et al. 2016). Such projects involve many aspects of risk, such as financial, political, market, economic, and supply chain risk: supply chain risks include all elements that can potentially disrupt supply chain flow. Complexities and uncertainties negatively affect the performance of the supply chain partners in project-oriented organizations (Rangel et al. 2015), hence supply chain risk management (SCRM) (Heckmann et al. 2015) is required to be addressed in green construction megaprojects (Balasubramanian and Shukla 2017).

SCRM’s purpose is to avoid possible negative outcomes of probable disruptions to reduce SC vulnerabilities (Rangel et al. 2015) and mitigate uncertainties and complexities to bring about resilience and competitive advantage (Thomé et al. 2016). For this purpose, SCRM identifies, assesses, and prioritizes risk factors, before the establishment of proper risk mitigation strategies to prevent failure or restore resilience (Fan and Stevenson 2018).

Questions outlining this research are as follows:

1. What are the priorities of supply chain oriented risk factors in the construction of green residential megaprojects for a case company in Iran?
2. What are the criteria representing the importance of these risk factors and which criterion is the most influential?
3. What are the implications of the results and what scenarios regarding the proposed criteria can be interpreted?

Some previous studies refer to the subject of green building projects’ risks, for example, Qin et al. (2016) identified and prioritized risk factors in green building projects, and Yang et al. (2016) identified, discerned the interrelations, and ranked project stakeholders’ key risk factors in green building projects, and Hwang et al. (2017) identified and ranked risk factors in various project phases of the green residential building.

From the supply chain perspective, the related literature suffers from the paucity of investigations on the construction projects’ risks, where Zou and Couani (2012) identified and ranked key supply chain risks of the development of green building projects and Rudolf and Spinler (2018) identified and ranked key risk factors of construction megaprojects.

There is a research gap in assessing and prioritizing green construction projects’ supply chain risks, especially using a systematic method. In this regard, systematic approaches such as Multi Criteria Decision Making (MCDM) and Multi Attribute Decision Making (MADM) methods have been used in the realm of operations research, engineering, and management science (Gal et al. 2013; Zavadskas et al. 2014). Among the compensatory MADM methods, Analytic Network Process (ANP) has been used in various disciplines such as risk assessment, as well (Kheybari et al. 2020). In terms of the project management domain, and specifically in the realm of construction projects, Dikmen et al. (2010) used DELPHI and ANP to assess and prioritize business failure risks in construction firms. Boateng et al. (2015) used ANP to prioritize risks at the construction stage of megaprojects. Valipour et al. (2015) used Fuzzy-ANP to prioritize freeway projects’ risks. And Karamoozian et al. (2019) used Decision Making Trial and Evaluation Laboratory (DEMATEL) and ANP to prioritize construction projects’ risks.

ANP has not yet been used to prioritize construction projects’ supply chain risks and its ability in quantifying the elements, calculating the feedback, and providing the row sensitivity analysis highly aligns with the objectives of the current research. Therefore, this research aims to illustrate a best practice of an ANP-based integrated method (Mu et al. 2020) to assess and prioritize, the formerly identified (Alamdari et al. 2021) supply chain oriented risk factors in the green construction of residential megaprojects.

Establishing the pairwise comparisons between a pair of child nodes concerning a control node, ANP analyzes the weights of each element in the system. The pairwise comparisons in the current research evaluate the importance of the risk factors known as the alternatives concerning four criteria specified, and evaluate the importance of the criteria concerning the alternatives, creating the feedback loop. The loop between the elements’ feedback provides a more realistic decision-making process. To establish pairwise comparisons between the alternatives, four criteria are used to represent the characteristics of each risk. The representative criteria are the impact, probability, manageability, and influence rate of each risk. The first two criteria are well known in risk management studies but the third and the fourth criteria introduced in this research offer novel value to the body of knowledge. The third criterion is manageability which is analogous to organizational resilience in the sense that: the more manageable the risks would be, the more project processes could be considered resilient. Manageability is used alongside impact and probability as quantifiers of pairwise comparisons, while the last criterion, influence rate, integrates the ISM into the ANP model regarding the driving powers of each alternative imported directly from a hierarchical model available in previous research. An expert panel comprised of four individuals was formed from the society of green construction of residential megaprojects, representing a focal construction company in Iran. Data analysis is administered in a Bell-shaped Fuzzy Set environment, while the utilization of fuzzy set theory provides an even more realistic transformation of qualitative judgments made by the expert panelists into crisp numbers, which then are used as input data in the SUPERDECISIONS software, that analyze pairwise comparisons and calculates the priorities. In addition, using the PYTHON coding language and the JUPITER platform, Row Sensitivity Analysis (RSA) is included to render scenarios interpreting the importance of the alternatives and criteria, and the effects of the criteria on the prominence of the alternatives.

The main contributions of the current research are our proposing and demonstrating a novel integrated Fuzzy-ANP-ISM method to prioritize supply chain oriented key risk factors, and applying it (for the first time to our knowledge) in a real setting of green construction of residential megaprojects. This required formulation of specific parameters for this setting. Another new contribution is the inclusion of an infrequently used supply chain risk property, namely manageability, other than the severity of impact and the probability of occurrence. And finally, utilization of a new fuzzy environment for the Fuzzy-ANP body of knowledge, especially for that the scope of the questionnaire remains intact, through the fuzzy-fi-cation.

The following are the highlights of the remaining sections. Section 2 briefly reviews the theoretical and the empirical literature of the subject matter. At the end of Sect. 2, the research gap and implications of the literature are rendered. Section 3 starts by briefly explaining the ANP method, and continues with defining the fuzzy environment used, row sensitivity analysis, and the application of ISM in the ANP model proposed. At the end of Sect. 3, there is the procedure, entitled as the general guidelines, by which the research has been administered and explained in more detail in Sect. 4. Section 4 describes the model, introduces the case study and the expert panelist, explains the tools and techniques required to execute the analyses or reproduce, and renders the results. Section 5 interprets and discusses the resulting output. And finally, Sect. 6 concludes the overall research, including the managerial and theoretical implications, limitations, and the scope of the further research.

## Literature review

### Green construction supply chain management

While the prime goal of supply chain management is to provide fluent functionality of processes to satisfy customers’ demands (Heckmann et al. 2015), GSCM, on the other hand, initially refers to a ranging scope of endeavors from reactive monitoring of general environmental management programs to more proactive practices (Srivastava 2007) mainly to enhance financial, operational, and environmental performance (Balon 2020). For instance, in the realm of construction projects, GSCM activities include green purchasing, green design, green manufacturing, green logistics, waste management, green operation, and end-of-life management (Badi and Murtagh 2019). However, while there is rising attention to aligning the construction industry with sustainable development goals, more proactive actions and proper global collaboration between the supply chain partners are required to attain long-term goals of sustainability (Asif et al. 2020; Tseng et al. 2019; Xie et al. 2022).

### Supply chain risk assessment terminologies

Those situations in the supply chain that might hinder organizations from continuing their businesses are known as supply chain risks (Heckmann et al. 2015). Supply chain literature also relates the definition of risk with concepts such as complexity, uncertainty, and resilience (Thomé et al. 2016). Some relatively new sources of uncertainty and risk factors such as COVID-19, increasingly challenging climate change risks and economic and political instability factors render some of early risk management processes as incomplete and unable to fully and effectively cope with the total supply chain risk picture facing executives. Early studies of risk management, including SCRM, were conducted in a more stable environment than in current global conditions. For example, COVID-19 has caused global shortages of many key ingredients ranging from timber to computer chips.

During the SCRM process, potential supply chain risks should be identified, assessed, prioritized, mitigated, recovered, and controlled. The assessment process discerns some characteristics of the risk factors that in prioritizing those risk factors would be required (Fan and Stevenson 2018; Ho et al. 2015; Rajagopal et al. 2017). Definitions suggested by the literature on SCRM (Pournader et al. 2020) and construction projects oriented risk management (Taroun 2014) repeatedly indicate the combination of specific components such as the likelihood of occurrence and impact of the consequences of operational, tactical, or strategic level failures or irregularities. However, additional criteria have also been introduced in the literature that can be useful in assessing supply chain risk events. In this respect, previous studies have discerned the impact of a triggering event by severity (or magnitude) and the consequential situation (or effect) imposed (Ritchie and Brindley 2007; Wagner and Bode 2008). Some other studies for example Giannakis and Papadopoulos (2016) and Abdelgawad and Fayek (2010) have considered another type of assessment criteria as the detectability of a risk event, which Ritchie and Brindley (2007) named the causal pathway. Abdelgawad and Fayek (2010) add another criterion namely controllability which is a time-based characteristic of the organizational ability to effectively influence risk sources before they lead to the occurrence of the risk event or controllability of the effect of a given risk event (Costantini et al. 2021; Heckmann et al. 2015). In the environment that increasingly is subjected to VUCA- Volatility, Uncertainty, Complexity, Ambiguity, (Bennett and Lemoine 2014; Mack et al. 2015), new and more powerful methods for dealing with project risk, especially involving the scale of mega projects, and the desire to create ‘Green’ outcomes, are required.

### Background of the prioritization of green supply chain risk factors in construction projects

Limited studies previously have prioritized supply chain oriented risk factors in construction projects. Rudolf and Spinler (2018) surveyed large-scale project managers and ranked the key risk factors by conducting a questionnaire, assessing the impact and probability of occurrence. Zou and Couani (2012) ranked supply chain risk factors of developing green building projects by eliciting industry experts’ opinions through a questionnaire survey. However, a few studies have prioritized green supply chain oriented risks in other industries, for example, Mangla et al. (2015) used fuzzy-AHP and sensitivity analysis. Song et al. (2017) used a rough-weighted DEMATEL method, and Rostamzadeh et al. (2018) used integrated fuzzy-TOPSIS-CRITIC. Three studies have prioritized risk factors in green building projects. Qin et al. (2016) used a questionnaire survey, Yang et al. (2016) administered the SNA method, and Hwang et al. (2017) used a questionnaire survey, ranking the risk factors based on the likelihood of occurrence and magnitude of impact.

### Summary and implication of the literature review

From the literature, two commonly used features, namely the severity of impact and the probability (frequency of occurrence), can be detected. A third feature, namely manageability, can be inferred, as a risk component related to controllability and resilience. Manageability measures the degree to which disruptions and or interruptions are controllable due to prevention or reactive mitigation. The last feature used in the current study, namely influence rate, leans on the term causal pathway. Influence rate is defined to compare the degree of influence each risk factor can inflict on other triggering events. No investigation has previously been conducted on the subject of prioritization of the supply chain oriented risk factors in the green construction of residential megaprojects, and a robust prioritization method would greatly contribute to the subject matter’s body of knowledge. Table 1 summarizes, from the literature and our conceptual development, the present state of the art in terms of these four risk features, each being described in terms of its meaning, maturity of concept and professional practice, and the costs of their ineffective treatment.

**Table 1:** Summarizing the risk assessment features

	**Probability**	**Severity of impact**	**Manageability**	**Influence**
Conceptual and practical interpretation of this element of risk management	How often and how likely is this risk element expected to be observed?	What is the expected magnitude of this risk element event?	To what extent can this risk element/ event be controlled, prevented, mitigated	How much does this risk element/ event impact by triggering other risks?
Maturity of core knowledge of this element	Has been considered a core element of traditional risk management for many decades, a range of industries (Pournader et al. 2020; Taroun 2014)	Has been considered a core element of traditional risk management for many decades, a range of industries (Ritchie and Brindley 2007; Wagner and Bode 2008)	Has more recently been acknowledged as key to effective risk management, but not yet universally conceptualized in many industries and contexts (Costantini et al. 2021; Heckmann et al. 2015)	Relatively nascent in concept, as risk factors have mostly been considered in isolation from each other, as individual contributors (Abdelgawad and Fayek 2010; Ritchie and Brindley 2007; Wagner and Bode 2008)
Maturity of professional practice of this element	Long considered a core element of risk management (Pournader et al. 2020; Taroun 2014)	Long considered a core element of risk management termed as impact (Pournader et al. 2020; Taroun 2014) Impact, then, was discerned by its severity and consequential situation (Ritchie and Brindley 2007; Wagner and Bode 2008)	Recently entering into leading edge practices in various industries, particularly the construction industry (Abdelgawad and Fayek 2010)	In early stages of consideration and immature in most risk prioritization practices (Abdelgawad and Fayek 2010)
Potential costs of ineffective treatment of this factor	Misapplication of resources by not taking frequency of occurrence sufficiently into account	Under-resourcing preparing for high severity risk events relative to low magnitude events	Risk elements that have difficulty or complexity of manageability may be under-resourced	Inter-risk elements may be only partially identified or fully unrecognized, hence underestimated
Costs of ineffective treatment in Green Construction	Under-accounting for frequency of occurrence of risks leads to poor risk mitigation through ineffective prioritization of high frequency risks, such as design error risks, construction material risks	Poor consideration of severity may compromise building’s green performance through insufficient attention paid to big ticket, high severity items such as insulation, material longevity	Prevention of ‘green’ performance problems may be under resourced or unplanned, leading to crisis or other problems post construction	Relationships between elements such as eco efficiency, longevity and material usage and wastage might be managed as separate issues, leading to sub-optimization and inferior performance outcome

## Research methodology

This section introduces the methodological development of the integrated problem-solving method utilized in the current research. The basic principles of each method, necessary for understanding the data analysis, are explained and a step-by-step procedure is determined (in Subsect. 3.5) based on which data gathering and analysis are conducted in Sect. 4.

### A brief introduction to the principles of the analytic network process (ANP)

ANP, developed by Saaty (1996), is a multi-criteria decision-making tool that deals with complicating pairwise comparisons and evaluates feedback between the elements in structured models. In brief, ANP assists the process of evaluations where mere hierarchic comparisons are not realistic enough. For example, it is more sensible to weigh up the criteria concerning the alternatives when prioritizing the alternatives, because the importance of the criteria may change each time, considering which alternative is selected. This cyclic connection treated in ANP is called feedback (Saaty 2004). Each pair of elements, namely child nodes, that are connected to a parent node should be compared individually, and decision panelists’ judgments accumulate to bring a solution to the whole model. The control criterion is a node that helps to think about the essence of comparison. The proposed qualitative measurement scale (Saaty 2013) consists of nine linguistic values which are then transformed into qualitative values, an ordinal scale with positive integers from 1 to 9. However, the more precise the experts’ judgments, the more accurate the resulting outcome. In that sense, to decrease the uncertainty of precision of transforming linguistic scales to numeric data, especially when dealing with human judgments, is utilized fuzzy logic, more on this is discussed in detail in Sect. 3.2. Nevertheless, the possible inconsistency, determined by consistency ratio (C.R.) regarding the transitivity property of the comparisons, should be less than 0.1, a deterministic rule that also coincides with statistical measurements given that the comparisons are consistent (Vargas 2008).

The C.R. is calculated through the following equation: C.R. = (C.I. / R.I.) ≤ 0.1 (Saaty 2013). C.I., consistency index, equals the principal eigenvalue of the local matrix minus the number of elements divided by the number of elements minus the degree of freedom which equals one, C.I. = (lambda max-n) ÷ (n-1); R.I., random index values, is computed using multiple simulations of randomly created comparison matrices and calculating the average of the consistency index.

Relative importance weights of the initial super-matrix, namely local priorities, can be calculated using different optimization methods such as the eigenvalue method, the least squares, the logarithmic least squares, or the weighted least squares (Golany and Kress 1993; Nishizawa and Takahashi 2009; Saaty and Vargas 1984). Filling the weight vectors regarding the particular child nodes related to any parent node, will form the unweighted matrix, and transforming it to column-wise stochastic matrix results in the weighted matrix, which means the probability of entries in each column will sum up to 1 and the resulting weighted matrix should be raised to a sufficiently significant power until it converges into a stable limit matrix. The limit matrix indicates the priority of each alternative or criterion (Saaty and Vargas 2013). The mathematical explanation of this step can be described as the equation below.

$$\begin{cases}{\text{W}}={\text{Lim}} {\text{W}}^{2{\text{k}}+1}\\ {\text{k}}\to \infty \end{cases}$$

### Proposed fuzzy set to evaluate linguistic data

The fuzzy set theory, introduced by Lotfi Zadeh (1965), discerns specific membership functions (MFs) for imprecisely defined classes of objects. Well-defined fuzzy sets are to deal with the imprecision triggered by the absence of sharply defined criteria of class membership and the values assigned range between zero and one. Fuzzy hybrid techniques of multi criteria decision-making, such as fuzzy ANP, are designed to assist the engineering professionals, particularly in construction project management, to handle uncertainties of the verbal statements or fuzzy ideas about the weights of the alternatives and or the criteria (Fayek 2020; Shafiee 2015).

A bell-shaped MF, one of the most commonly used shapes of type-1 fuzzy sets (Mendel 2017), which is considered more appropriate in dealing with linguistic variables (Maturo and Fortuna 2016), is conducted in the current research and follows the below-mentioned function. Where the symbol $\upmu$_A(x),_ represents MF, the letter d is the factor that controls the width of the curve, the letter x represents the horizontal axis and the letter c is the center of the corresponding bell graph. Using the one expert’s direct intuitionistic judgment method (Abdelgawad and Fayek 2012; Klir and Yuan 1995) the exact shape of the bell-graph was elicited, which led to specifying variable c to be equivalent to ten (c = 10).

$${\mu }_{{\text{A}}({\text{x}})}=1/\left[1+{\text{d}}{\left({\text{x}}-{\text{c}}\right)}^{2}\right]$$

Specific fuzzy numbers can be calculated from the chosen fuzzy function, discretely, to each verbal statement and after algebraic summation of all the experts’ judgments cast on a specific comparison, using the Center of Gravity (SG) method the accumulated resulting fuzzy number is defuzzified to a crisp number. The discrete form of the SG method follows the equation below.

$$a=\Sigma {\mu }_{\text{A}}\left({\text{xi}}\right).{\text{xi}}/\Sigma {\mu }_{\text{A}}\left({\text{xi}}\right)$$

The letter a, represents a specified distance of the center of gravity from the vertical axis; the symbol $\upmu$_A(x),_ represents MF; the symbol xi is any point on the horizontal axis. It is worth recalling that in the ANP questionnaire i = 1, 2, 3, …, 9. The resulting fuzzy numbers equivalent to each verbal statement are calculated using the software MATLAB and are shown in Table 2.

**Table 2:** The fuzzy sets assigned to the linguistic values

The linguistic values	The membership functions								
*equally as important as*	1	0.0909	0.0244	0.0110	0.0062	0.0040	0.0028	0.0020	0.0016
*equally to moderately more important than*	0.0909	1	0.0909	0.0244	0.0110	0.0062	0.0040	0.0028	0.0020
*moderately more important*	0.0244	0.0909	1	0.0909	0.0244	0.0110	0.0062	0.0040	0.0028
*moderately to strongly more important*	0.0110	0.0244	0.0909	1	0.0909	0.0244	0.0110	0.0062	0.0040
*strongly more important than*	0.0062	0.0110	0.0244	0.0909	1	0.0909	0.0244	0.0110	0.0062
*strongly to very strongly more important than*	0.0040	0.0062	0.0110	0.0244	0.0909	1	0.0909	0.0244	0.0110
*very strongly more important than*	0.0028	0.0040	0.0062	0.0110	0.0244	0.0909	1	0.0909	0.0244
*very strongly to extremely more important than*	0.0020	0.0028	0.0040	0.0062	0.0110	0.0244	0.0909	1	0.0909
*extremely more important*	0.0016	0.0020	0.0028	0.0040	0.0062	0.0110	0.0244	0.0909	1
The corresponding fuzzy steps	1st	2nd	3rd	4th	5th	6th	7th	8th	9th

The sample example below illuminates the calculation required to convert experts’ linguistic responses into a crisp number. To bring light on the subject matter, consider in a given expert panel consisting of four individuals, the responses were ‘moderately more important than Second’; ‘strongly more important than’; ‘very strongly more important than’; ‘extremely more important’. The corresponding fuzzy sets would be as shown in Table 3.

**Table 3:** The fuzzy sets assigned to the linguistic values

The first expert’s response	0.0244	0.0909	1	0.0909	0.0244	0.0110	0.0062	0.0040	0.0028
The second expert’s response	0.0062	0.0110	0.0244	0.0909	1	0.0909	0.0244	0.0110	0.0062
Third third expert’s response	0.0028	0.0040	0.0062	0.0110	0.0244	0.0909	1	0.0909	0.0244
The fourth expert’s response	0.0016	0.0020	0.0028	0.0040	0.0062	0.0110	0.0244	0.0909	1
The corresponding fuzzy step	1st	2nd	3rd	4th	5th	6th	7th	8th	9th

To produce an average out of the fuzzy sets resulting, the average of the values allotted to each step should be calculated. The resulting would be a fuzzy number, as shown in Table 4.

**Table 4:** the average fuzzy number resulted

The resulting fuzzy number	0.0088	0.0270	0.2584	0.0492	0.2638	0.0510	0.2638	0.0492	0.2584	**1.23**
The corresponding fuzzy step	1st	2nd	3rd	4th	5th	6th	7th	8th	9th	Sum

The calculations should proceed with multiplying each step’s value by its step number. The resulting would be a weighted fuzzy number, as shown in Table 5.

**Table 5:** The weighted fuzzy number

The resulting fuzzy number	0.0088	0.0540	0.7752	0.1968	1.3187	0.3057	1.8462	0.3936	2.3256	**7.22**
The corresponding fuzzy step	1st	2nd	3rd	4th	5th	6th	7th	8th	9th	Sum

Finally, dividing the summation of the values of the weighted fuzzy number by the summation of the average fuzzy number, the ultimate crisp value allocated to a specific pairwise comparison results. In this particular case, the resulting crisp value would be: 7.22 ÷ 1.23 = 5.87.

### Row sensitivity analysis

To explain the need for sensitivity analysis when prioritizing risk factors, one might consider two schools of risk, risk as a subjective perception and risk as an objective construct with each requiring different risk management and mitigation strategies (Zhang 2011). For instance, in construction megaprojects, structural or known uncertainties are avoidable mainly through the reduction of complexity (Giezen 2012), while unpredictable uncertainties require crisis management (Lehtiranta 2011). Therefore, to attain agility and flexibility, managers are advised to be aware of inert unmanaged assumptions and or changing conditions of uncertainty in risk management practices where they can utilize predictive, adaptive, or hybrid methodologies of risk control (Costantini et al. 2021).

Row sensitivity analysis (RSA) developed by Adams (2014) is a calculation technique for ANP models that provides scenarios in light of modifying the weights of the nodes. Changing the importance of a particular node, while modifying the weights of the remaining nodes proportionately concerning the original structure of the weighted matrix (described in Sect. 3.1), equips the decision-makers with the foresight of prediction and planning (theoretical explanations and a sample calculation can be found in the Appendix section).

### The application of interpretive structural modeling (ISM)

ISM introduced by Warfield (1974) is an interactive learning process that provides structured hierarchies, reflecting the flow of the contextual relationship permeated between a set of elements (Farris and Sage 1975; Malone 1975; Warfield 1976). The systematic logical thinking administered by ISM is a widely used technique in multiple disciplines that equips decision-makers with a better comprehension of complex interdependencies between the elements of a system regarding the potential influence they may have on each other (Cherrafi et al. 2017; Hughes et al. 2020; Kumar and Goel 2021). During the ISM procedure (Jharkharia and Shankar 2004) some quantitative values namely driving powers can be assessed for each particular element, representing the number of elements it can directly or indirectly influence. For instance, in the context of risk management, the more a risk factor can act as a triggering source for the concomitant risks in a project, the more it can incur negative effects on the success of the whole project. However, these quantitative values can be interpreted as the degree of importance concerning their driving power, a criterion introduced in the current research as the influential rate, described in detail in Sect. 4.

### Proposed integrated ISM-FANP

**Fig. 1:**
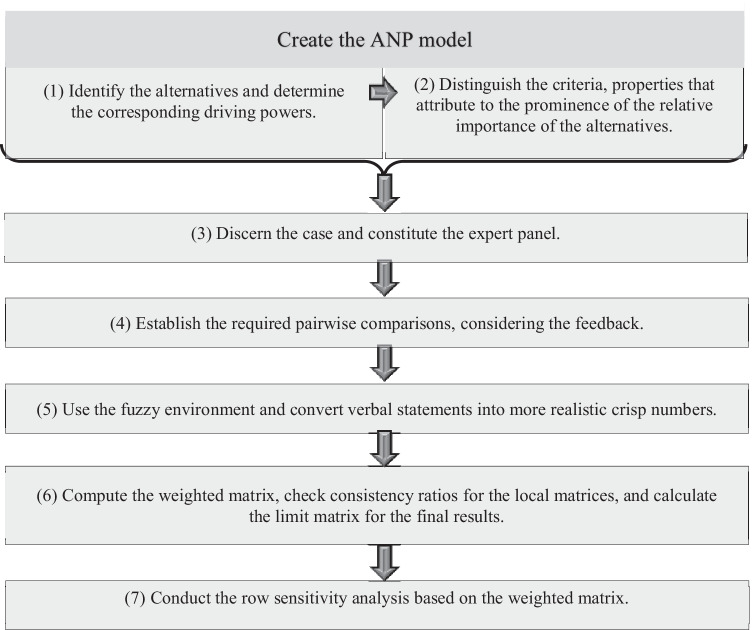
the proposed research guideline

Effective supply chain risk assessment strategies, in general, comprise two parts (Fan and Stevenson 2018), and can be based on objective physical data or experts’ perceptive judgments and the establishment of scenarios (Cohen and Kunreuther 2007). One component is to establish the interrelationships between risk factors. Structuring the interrelationships between the risk factors possibly leads to the determination of the criticality of those that trigger the other risks (Venkatesh et al. 2015). The other component is to prioritize those risk factors. The following general steps shown in Fig. 1 are indicating the general guideline proposed in the current study to prioritize supply chain oriented risk factors.

The integrated method proposed, primarily uses ANP in a fuzzy environment to prioritize some alternatives of key risk factors. A comprehensive literature review determined the criteria required to establish pairwise comparisons based on experts’ judgments. In addition, ISM identifies the key risk factors, namely the alternatives. And the driving powers calculated for each alternative were integrate into the ANP model determining the fourth criterion and their corresponding weights. Finally, row sensitivity analysis determines the stability of the results and provides possible scenarios.

## The proposed model and data analysis

Section four comprises six sub-sections. At first, the introduction of the alternatives and the criteria, the main components of the ANP model, is provided, followed by the description of the case and the introduction to demographic profiles of the panelists in the second sub-section. The third sub-section explains the process of pairwise comparisons. The fourth sub-section explains the fuzzy data analysis in detail. Results of the weighted matrix, consistency ratios, the limit matrix, and lastly the priorities of the criteria and alternatives are rendered in the fifth sub-section. Finally, the last sub-section renders the outputs of the row sensitivity analysis.

### The ANP model

The proposed system consists of two types of components, namely the alternatives and the criteria. Following the subject matter, the alternatives are supply chain oriented key risk factors in the green construction of residential megaprojects, and the criteria are the features that assist in discerning the relative importance of the alternatives. The alternatives are imported from a former study (Alamdari et al. 2021) which has identified twelve all-inclusive items, through comprehensive literature review and semi-structured interview sessions with an international diverse panel of fifteen experts. In addition, the study has assessed the triggering interrelations between the alternatives, using the ISM method through a three-round DELPHI process with six individual diverse industry experts. The number of elements that each alternative can trigger is the driving power of that particular alternative. The alternatives of risk events and the driving powers (D.P.) attributed are represented in Table 6. On other hand, four criteria namely, impact, probability, manageability, which have been discussed in Sect. 2, and the influence rate, which has been discussed in Subsects. 2.3 and 3.4, are included in the ANP model. A brief description of the criteria used is represented in Table 7.

**Table 6:** The alternatives of risk events and the attributed D.P. *(*Alamdari et al. 2021*)*

Code	Alternative	Description	D.P
R1	Key green supplier failure	Insufficient provision of environmentally friendly tasks such as material, knowledge, information, goods, equipment, or services regarding, time, price, and quality	8
R2	Unavailability of raw green materials and equipment	Unavailability of green raw materials and equipment, tools, or apparatus that minimize energy consumption, reduce pollution, and optimize process time, regarding quality, speed, and flexibility	8
R3	Lack of commitment to the implementation of green practices	Managers and or employees, while do not acknowledge the benefits, they refrain from initiating or implementing some specific green practices	8
R4	Misfit of corporate cultures	Non-supportive corporate cultures and or sub-cultures that hamper the project from achieving its greening goals and objectives	10
R5	Unsatisfactory green information/knowledge and deficiency in the level of green process technology	Failures in providing requisite green information technology systems, process design, materials, information, and operational ventures	4
R6	Failure to reach the quality expected of the building	Inadequate perceived quality of the final product during the project lifecycle, end of use, maintenance, and recycling period, regarding health, safety, efficiency, and comfort	1
R7	Logistic coordination complexity and green supply chain configuration error	Errors related to process management such as transportation, manufacturing, or staff, technical executive operations regarding green logistic activities, and supply chain configuration regarding facility role and location, capacity, or market	2
R8	Poor planning (inaccurate green project goals, scope, scheduling), execution, and control	Logistic-oriented errors related to green activities, regarding budgeting, resource allocation, expenditure estimation, responsibility appointment, and project progress evaluation	3
R9	Scarcity of experts, Experienced and skilled labor, in the context of green construction	The paucity of skilled labor and expertise, regarding green construction obligations and procedures, work instructions, and prevalent regulations and policies	9
R10	No mutual commitment, collaboration, and teaming	Improper communication between green supply chain stakeholders and project members, regarding trust, honesty, and collaboration	8
R11	Moral hazards	Risks taken irrationally to benefit specific parties while, otherwise, the detriments of losses would be shared among the whole parties involved	9
R12	Key customers’ reluctance in support of green initiatives or green products and services	Inability to attract key customers to use the final product developed and support the implementation of green activities in residential megaprojects	9

**Table 7:** Description of the criteria

Criterion	Description
Impact	The severity and the consequential situation imposed by the triggering event
Probability	The frequency of occurrence of the triggering event
Manageability	The degree to which disruptions and or interruptions are controlled due to prevention or reactive mitigation
Influence rate	The degree of influence each risk factor can inflict on other triggering events

**Fig. 2:**
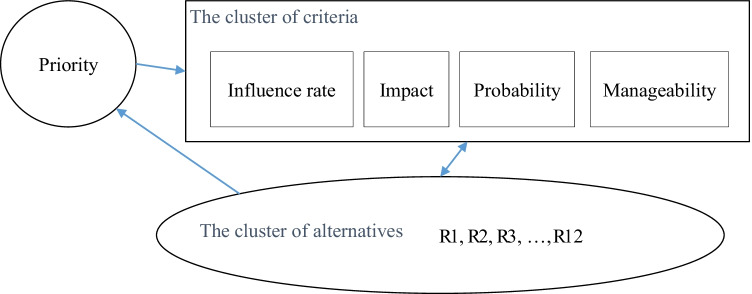
The figurative ANP model

However, the ANP model proposed has two clusters, alternatives, and criteria, and the ultimate goal is the priority of the alternatives. The schematic ANP model which was formed in the SUPERDECISIONS software is represented in Fig. 2. The connections between the nodes are discussed in detail in Sect. 4.3.

### Introduction to the case and the expert panel

The PARDISAN company, located in Iran, is a consult, research, and design-oriented service provider in multiple fields such as railways, metros, damps, ports, bridges, and buildings. The company has been involved in several large-scale building projects, has attained ISO 14000, and is considered the country’s one of the few leading green building project executors. Its four individual senior managers constitute the expert panel, experienced and knowledgeable professional engineers that had senior administrative roles in the company. The profile of the expert panelists is shown in Table 8.

**Table 8:** The profile of the panelists

	**Education**	**Position**	**Experience**	**Country**
1^st^ panelist	Ph.D.—Civil Engineering/ Structural designer	Senior manager	20 Yrs. consultant/ University lecturer	Iran
2^nd^ panelist	Masters – Civil Engineering	Senior LEED examiner	10 Yrs. consultant/ university lecturer	Iran/UK
3^rd^ panelist	Ph.D. – Geotechnical Engineering	Senior manager	10 Yrs. consultant/ Project coordinator	Iran
4^th^ panelist	Masters—Geotechnical Engineering	Senior manager	10 Yrs. consultant	Iran

### Establishment of the pair-wise comparisons

The purpose of the pair-wise comparisons is to measure the relative importance between the pairs of nodes concerning parent nodes specified in the system. All the measurements accumulate and result in priorities. Besides that the alternatives should be compared concerning criteria, the relative importance of the criteria may change concerning different alternatives either, hence the feedback loop in the ANP method is requisite. Expert panelists answered sixteen sub-questions and overall made two hundred and forty comparisons, each. However, the main general quotation states as: which of the pair of the nodes given is more important concerning the given control criterion? Sub-questions provide a control criterion and a cluster of elements that should be compared. Table 9 illustrates the components of the sub-questions.

**Table 9:** The components of the sub-questions

No	Parent node given	The given cluster of elements	No	Parent node given	The given cluster of elements
*Q.1*	Node R1	Cluster of criteria	*Q.9*	Node R9	Cluster of criteria
*Q.2*	Node R2	Cluster of criteria	*Q.10*	Node R10	Cluster of criteria
*Q.3*	Node R3	Cluster of criteria	*Q.11*	Node R11	Cluster of criteria
*Q.4*	Node R4	Cluster of criteria	*Q.12*	Node R12	Cluster of criteria
*Q.5*	Node R5	Cluster of criteria	*Q.13*	Node Impact	Cluster of alternatives
*Q.6*	Node R6	Cluster of criteria	*Q.14*	Node Probability	Cluster of alternatives
*Q.7*	Node R7	Cluster of criteria	*Q.15*	Node Manageability	Cluster of alternatives
*Q.8*	Node R8	Cluster of criteria	*Q.16*	Node Priority	Cluster of criteria

The expert panelists then select the degree to which a node is prevailing over the other or can choose the equivalent importance. The figurative sample of pair-wise weighting is depicted in Table 10.

**Table 10:**
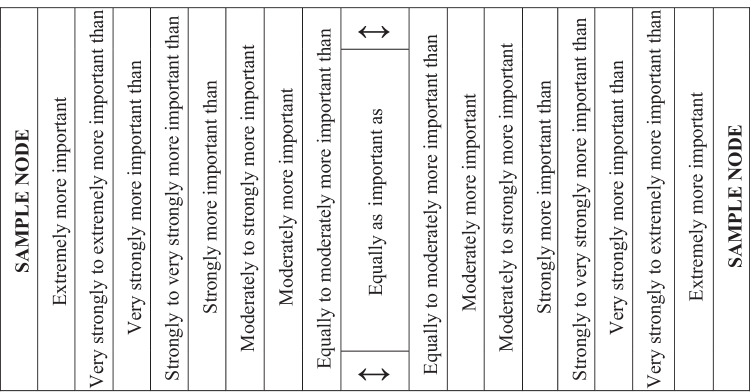
The pair-wise comparison’s nine scale verbal statements spectrum

### Calculation of the linguistic values using the bell-shaped fuzzy set selected

For each pair-wise comparison, four verbal evaluations were presented, by the four expert panelists, that should be aggregated. Using the membership functions attributed to each verbal statement (please refer to Table 2), each specified step’s corresponding numeric values should be algebraically accumulated. To defuzzify the resulting fuzzy number, the integer at each fuzzy step should be multiplied by the corresponding step number. For example, an integer at step three should be multiplied by 3, or an integer at step six should be multiplied by 6. The summation of the newly resulting fuzzy number’s membership values divided by the summation of the originally aggregated fuzzy number’s membership values, results in the crisp value, desired. The resulting crisp value is the relative importance weight of the node that prevails over the other. Utilizing the MICROSOFT EXCEL, all the verbal pair-wise comparisons were calculated into crisp values.

### Data analysis and results

The resulting two hundred and forty crisp values, the number of pair-wise comparisons as mentioned in Subsect. 4.3, are considered as the input for the SUPERDECISIONS software that creates the super-matrix and local matrices and calculates weighted matrix and limit matrix. The resulting weighted matrix and limit matrix are shown respectively in Tables 11 and 12.

**Table 11:** The weighted matrix

	R1	R2	R3	R4	R5	R6	R7	R8	R9	R10	R11	R12	Impact	Influence rate	Manageability	Probability	Priority
R1	**0**	**0**	**0**	**0**	**0**	**0**	**0**	**0**	**0**	**0**	**0**	**0**	**0.05**	**0.10**	**0.06**	**0.03**	**0**
R2	**0**	**0**	**0**	**0**	**0**	**0**	**0**	**0**	**0**	**0**	**0**	**0**	**0.09**	**0.10**	**0.10**	**0.10**	**0**
R3	**0**	**0**	**0**	**0**	**0**	**0**	**0**	**0**	**0**	**0**	**0**	**0**	**0.02**	**0.10**	**0.03**	**0.02**	**0**
R4	**0**	**0**	**0**	**0**	**0**	**0**	**0**	**0**	**0**	**0**	**0**	**0**	**0.02**	**0.12**	**0.04**	**0.01**	**0**
R5	**0**	**0**	**0**	**0**	**0**	**0**	**0**	**0**	**0**	**0**	**0**	**0**	**0.07**	**0.05**	**0.05**	**0.05**	**0**
R6	**0**	**0**	**0**	**0**	**0**	**0**	**0**	**0**	**0**	**0**	**0**	**0**	**0.23**	**0.01**	**0.18**	**0.21**	**0**
R7	**0**	**0**	**0**	**0**	**0**	**0**	**0**	**0**	**0**	**0**	**0**	**0**	**0.02**	**0.02**	**0.03**	**0.02**	**0**
R8	**0**	**0**	**0**	**0**	**0**	**0**	**0**	**0**	**0**	**0**	**0**	**0**	**0.04**	**0.03**	**0.06**	**0.08**	**0**
R9	**0**	**0**	**0**	**0**	**0**	**0**	**0**	**0**	**0**	**0**	**0**	**0**	**0.14**	**0.11**	**0.13**	**0.13**	**0**
R10	**0**	**0**	**0**	**0**	**0**	**0**	**0**	**0**	**0**	**0**	**0**	**0**	**0.02**	**0.10**	**0.03**	**0.04**	**0**
R11	**0**	**0**	**0**	**0**	**0**	**0**	**0**	**0**	**0**	**0**	**0**	**0**	**0.02**	**0.11**	**0.03**	**0.03**	**0**
R12	**0**	**0**	**0**	**0**	**0**	**0**	**0**	**0**	**0**	**0**	**0**	**0**	**0.23**	**0.11**	**0.20**	**0.23**	**0**
Impact	**0.34**	**0.25**	**0.34**	**0.24**	**0.33**	**0.32**	**0.27**	**0.04**	**0.16**	**0.08**	**0.16**	**0.36**	**0**	**0**	**0**	**0**	**0.31**
Influence rate	**0**	**0**	**0**	**0**	**0**	**0**	**0**	**0**	**0**	**0**	**0**	**0**	**0**	**0**	**0**	**0**	**0.31**
Manageability	**0.04**	**0.04**	**0.04**	**0.04**	**0.04**	**0.05**	**0.04**	**0.22**	**0.16**	**0.32**	**0.16**	**0.06**	**0**	**0**	**0**	**0**	**0.06**
Probability	**0.10**	**0.20**	**0.10**	**0.20**	**0.11**	**0.12**	**0.17**	**0.22**	**0.16**	**0.08**	**0.16**	**0.06**	**0**	**0**	**0**	**0**	**0.29**
Priority	**0.50**	**0.50**	**0.50**	**0.50**	**0.50**	**0.50**	**0.50**	**0.50**	**0.50**	**0.50**	**0.50**	**0.50**	**0**	**0**	**0**	**0**	**0**

**Table 12:** the limit matrix

	R1	R2	R3	R4	R5	R6	R7	R8	R9	R10	R11	R12	Impact	Influence rate	Manageability	Probability	Priority
R1	**0.02**	**0.02**	**0.02**	**0.02**	**0.02**	**0.02**	**0.02**	**0.02**	**0.02**	**0.02**	**0.02**	**0.02**	**0.02**	**0.02**	**0.02**	**0.02**	**0.02**
R2	**0.04**	**0.04**	**0.04**	**0.04**	**0.04**	**0.04**	**0.04**	**0.04**	**0.04**	**0.04**	**0.04**	**0.04**	**0.04**	**0.04**	**0.04**	**0.04**	**0.04**
R3	**0.01**	**0.01**	**0.01**	**0.01**	**0.01**	**0.01**	**0.01**	**0.01**	**0.01**	**0.01**	**0.01**	**0.01**	**0.01**	**0.01**	**0.01**	**0.01**	**0.01**
R4	**0.02**	**0.02**	**0.02**	**0.02**	**0.02**	**0.02**	**0.02**	**0.02**	**0.02**	**0.02**	**0.02**	**0.02**	**0.02**	**0.02**	**0.02**	**0.02**	**0.02**
R5	**0.02**	**0.02**	**0.02**	**0.02**	**0.02**	**0.02**	**0.02**	**0.02**	**0.02**	**0.02**	**0.02**	**0.02**	**0.02**	**0.02**	**0.02**	**0.02**	**0.02**
R6	**0.07**	**0.07**	**0.07**	**0.07**	**0.07**	**0.07**	**0.07**	**0.07**	**0.07**	**0.07**	**0.07**	**0.07**	**0.07**	**0.07**	**0.07**	**0.07**	**0.07**
R7	**0.01**	**0.01**	**0.01**	**0.01**	**0.01**	**0.01**	**0.01**	**0.01**	**0.01**	**0.01**	**0.01**	**0.01**	**0.01**	**0.01**	**0.01**	**0.01**	**0.01**
R8	**0.02**	**0.02**	**0.02**	**0.02**	**0.02**	**0.02**	**0.02**	**0.02**	**0.02**	**0.02**	**0.02**	**0.02**	**0.02**	**0.02**	**0.02**	**0.02**	**0.02**
R9	**0.05**	**0.05**	**0.05**	**0.05**	**0.05**	**0.05**	**0.05**	**0.05**	**0.05**	**0.05**	**0.05**	**0.05**	**0.05**	**0.05**	**0.05**	**0.05**	**0.05**
R10	**0.02**	**0.02**	**0.02**	**0.02**	**0.02**	**0.02**	**0.02**	**0.02**	**0.02**	**0.02**	**0.02**	**0.02**	**0.02**	**0.02**	**0.02**	**0.02**	**0.02**
R11	**0.02**	**0.02**	**0.02**	**0.02**	**0.02**	**0.02**	**0.02**	**0.02**	**0.02**	**0.02**	**0.02**	**0.02**	**0.02**	**0.02**	**0.02**	**0.02**	**0.02**
R12	**0.08**	**0.08**	**0.08**	**0.08**	**0.08**	**0.08**	**0.08**	**0.08**	**0.08**	**0.08**	**0.08**	**0.08**	**0.08**	**0.08**	**0.08**	**0.08**	**0.08**
Impact	**0.17**	**0.17**	**0.17**	**0.17**	**0.17**	**0.17**	**0.17**	**0.17**	**0.17**	**0.17**	**0.17**	**0.17**	**0.17**	**0.17**	**0.17**	**0.17**	**0.17**
Influence rate	**0.06**	**0.06**	**0.06**	**0.06**	**0.06**	**0.06**	**0.06**	**0.06**	**0.06**	**0.06**	**0.06**	**0.06**	**0.06**	**0.06**	**0.06**	**0.06**	**0.06**
Manageability	**0.05**	**0.05**	**0.05**	**0.05**	**0.05**	**0.05**	**0.05**	**0.05**	**0.05**	**0.05**	**0.05**	**0.05**	**0.05**	**0.05**	**0.05**	**0.05**	**0.05**
Probability	**0.11**	**0.11**	**0.11**	**0.11**	**0.11**	**0.11**	**0.11**	**0.11**	**0.11**	**0.11**	**0.11**	**0.11**	**0.11**	**0.11**	**0.11**	**0.11**	**0.11**
Priority	**0.20**	**0.20**	**0.20**	**0.20**	**0.20**	**0.20**	**0.20**	**0.20**	**0.20**	**0.20**	**0.20**	**0.20**	**0.20**	**0.20**	**0.20**	**0.20**	**0.20**

In each column, the allocated normalized weight of a particular element, located at the left wing of the matrix, can be identified and since the values are normalized, each column adds up to 1. The zero values indicate that there has not been a pairwise comparison for those specific elements with respect to the specified elements located at the top of the matrix.

The limit matrix identifies the final priority of the elements while the values of each row identifies the stabilized weight allocated to the corresponding element located at the left wing of the matrix.

**Fig. 3:**
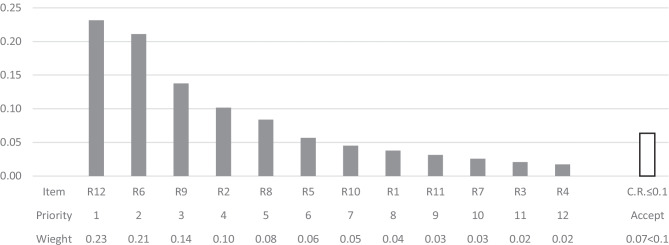
The cluster priorities of the alternatives with respect to the criterion Probability

**Fig. 4:**
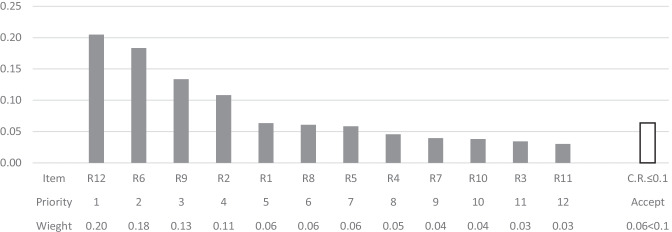
The cluster priorities of the alternatives with respect to the criterion Manageability

**Fig. 5:**
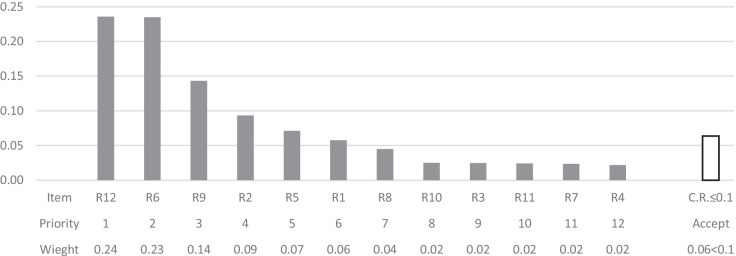
The cluster priorities of the alternatives with respect to the criterion Impact

**Fig. 6:**
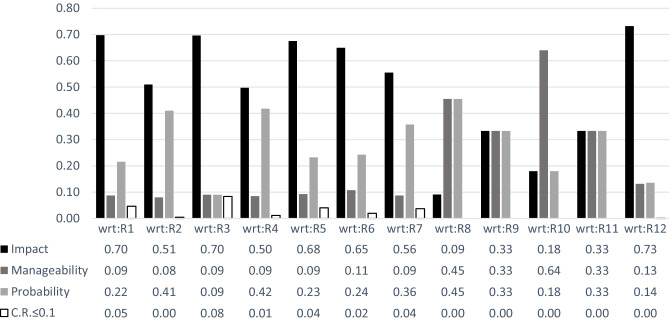
The cluster priorities with respect to the alternatives

The following diagrams depict local priorities, overall priorities, and their corresponding weights. The resulting cluster priorities of the alternatives, based on the normalized local weights of the local matrices, with respect to the criteria probability, manageability, and impact are shown respectively in Figs. 3, 4, and 5. On the other hand, the resulting normalized cluster priorities of the criteria, based on the normalized local weights of the local matrices, with respect to the alternatives are shown respectively in Fig. 6. All the inconsistencies calculated, C.R., are less than 0.1, therefor pair-wise comparisons are considered acceptable.

Results indicate that the first four high priority alternatives namely, R12, R6, R9, and R2 remain consistent with respect to only one criterion at a time. Changes can be seen in cluster priority of the alternatives for the less important alternatives. However, the degree of importance of each criterion may be different. Hence, the information provided in Fig. 6 is to shed light on the fact that, with respect to different alternatives, the importance of each criterion can be different. For instance, the criterion impact has received the highest normalized weight of 0.70 with respect to the alternative R1, while it has received the lowest normalized weight of 0.09 with respect to the alternative R8, and all three criteria engaged in pair-wise comparisons have received an equivalent normalized weight of 0.33 with respect to the alternatives R9 and R11.

**Fig. 7:**
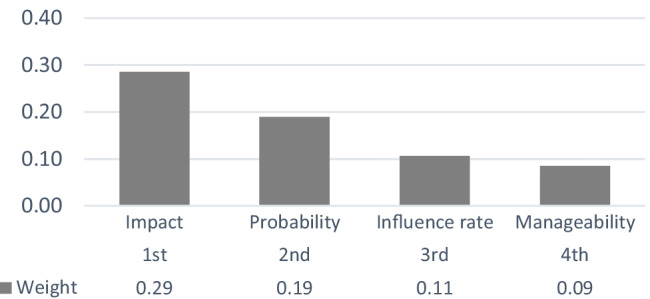
Priorities of the criteria

**Fig. 8:**
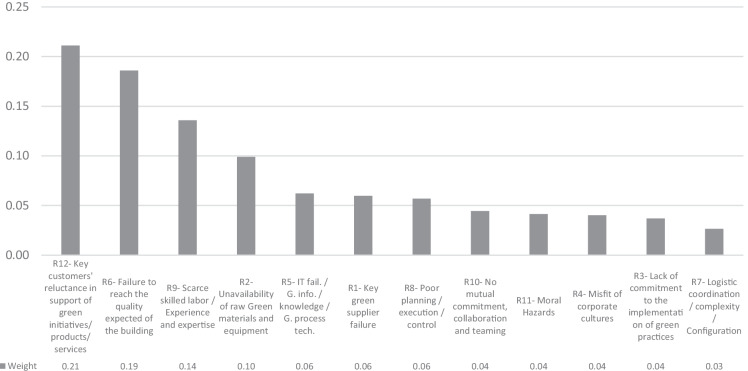
Priorities of the alternatives

The findings lead to the conclusion that in order to prioritize the alternatives of risk events in the proposed model, the feedback loop were rightfully being entered into the calculations. Therefore, the overall priorities of the alternatives will render more reliable outcomes, taking into consideration the variable degree of importance of the criteria. The overall priorities of the criteria and priorities of the alternatives based on their corresponding normalized weights derived from the limit matrix (Table 12) are shown respectively in Figs. 7 and 8.

The final results indicate that the most contributory criterion in determining the prominence of the key risk factors is severity of impact followed by probability of occurrence (please refer to Fig. 7). However, both influence rate and manageability have also received considerable importance weights, so should not be neglected by all means. Results also indicate the prominence of R12, R6, R9, and R2 (please refer to Fig. 8). It is worth mentioning that the final priorities of the alternatives found in Fig. 8 depend on then prevalent circumstances and the specific organizational situation of the case under study. However, the final results could vary under different and or changing conditions or could even be altered by conducting particular deeds and or practices. The following sub-section takes into consideration some probable changes in the priority of the alternatives given that the weights of the criteria could be different in various scenarios.

### Row sensitivity analysis

**Fig. 9:**
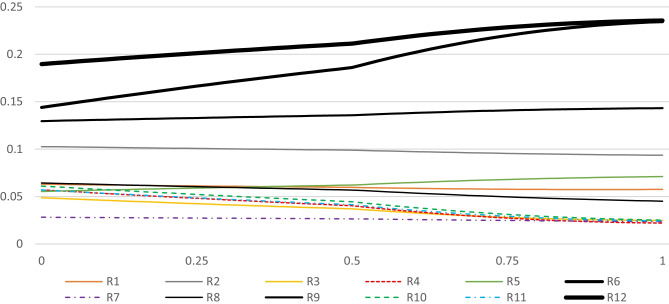
Sensitiveness of the alternatives regarding the criterion Impact

**Fig. 10:**
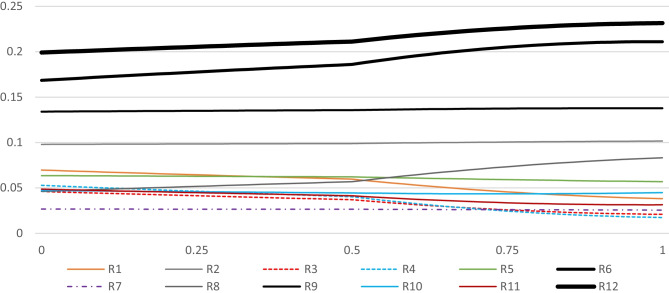
Sensitiveness of the alternatives regarding the criterion Probability

**Fig. 11:**
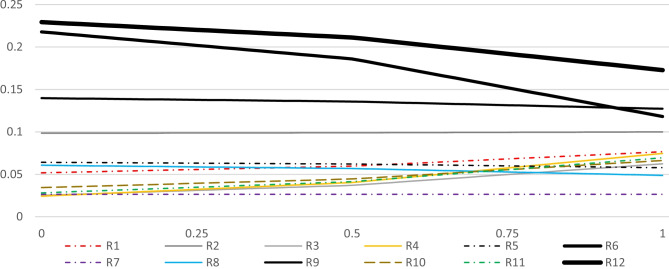
Sensitiveness of the alternatives regarding the criterion influence rate

**Fig. 12:**
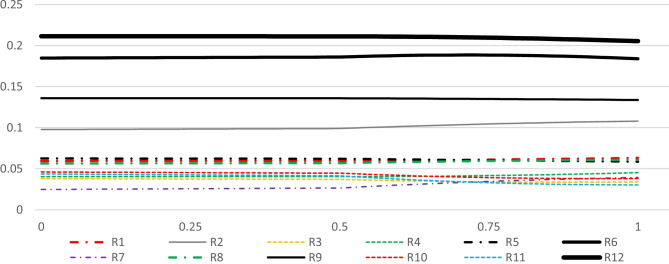
Sensitiveness of the alternatives regarding the criterion Manageability

**Fig. 13:**
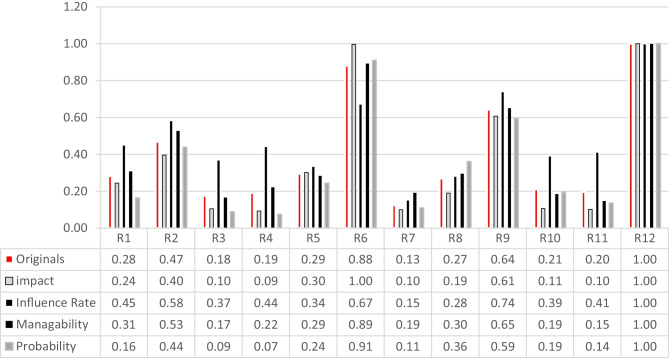
Perspective sensitivity analysis showing new scores

To avoid tedious calculations and data management, PYTHON coding language was used on the JUPITER platform (Adams 2022). The input required would be a weighted matrix, which in the current research the required data is shown in Table 11, to acquire the results of the row sensitivity analysis. Figures 9, 10, 11, and 12 illustrate the sensitiveness of the alternatives concerning the weight changes in each criterion at a time. Figure 13 renders another aspect, the perspective analysis, based on the idealized scores of the alternatives compared to their original values, given that the corresponding criterion would be the most important one.

It is worth mentioning that each risk factor is distinguished by a varying shape, where their corresponding symbolic legends are provided beneath each spectrum. The overall ranks of the alternatives are shown at the mid-point of each spectrum. Moving to the right in Fig. 9 considers the proportionate increase in the importance of the criterion impact in comparison with the other criteria, and moving to the left, considers the proportionate decrease in the importance of impact. In the same manner, Figs. 10, 11, and 12 respectively consider the proportionate increase in the importance of the criterion probability, influence rate, and manageability. The closer to the mid-point a weight change or rank change occurs in terms of the alternatives, the more is the degree of sensitivity of the specified risk factor regarding the corresponding criteria. At those points that any pair of lines get closer to each other, the closer gets their corresponding importance weights.

Based on the results, it is expected that the alternatives’ ranks do not change when the importance of the impact of the risk events increases. However, the difference in the importance weights between the alternatives can deviate. R12 and R6 become more and more important, R9 remains considerably important, but while R2, R5, R1, and R8 remain relatively important, the rest of the alternatives plummet. When the importance of the impact decreases, some changes in the ranks can be seen, especially when impact is the least important criterion. The relative importance of the most prominent alternatives, namely R12, R6, R9, and R2, do not change drastically. In terms of the less important alternatives, their relative importance shrinks and some changes in their rank order is detectable. On the other hand, when the priority weight of the criterion probability increases, the importance of R8 surges, altering its rank from eighth to fifth, not very far from the mid-point of the spectrum. In contrast, when the importance weight of the criterion influence increases, R8’s rank drops to the eleventh place. While almost all of the less important alternatives rise in their importance, to some extent, the most prominent change happens to be the rank exchange between R6 and R9. Finally, increase in the importance of the criterion manageability, incurs some trivial changes in the rank order of the least important alternatives. R7, for the first time, does not rank the least important alternative, but still, its relative importance is not significant compared to others.

The perspective sensitivity analysis, illustrated in Fig. 13, provides the required information to make comparisons between the current situation’s resulting priorities of the risk factors with those situations in which each of the criteria could receive the highest importance weight in the final weighted matrix. In other words, perspective sensitivity analysis identifies the utmost sensitiveness of each risk factor when each criterion is supposed to receive the highest importance weight. Therefore, the most crucial changes in the priority weights of the risk factors can be discerned way easier. The original values indicate the idealized scores of the risk factors at their current situation, in which the highest score equals to 1. Four new idealized scores are likely to be given to each risk factor concerning the four criteria. The higher the deviation between the current and a new score, the higher the specified risk factor’s sensitivity regarding the corresponding criterion. For example, R4 exhibits a considerable degree of sensitivity: the current idealized score of R4 is 0.19 but when impact reaches the utmost, its (R4) new idealized score would decrease and equal to 0.09, and when influence reaches the utmost, its score, this time, would increase and equal to 0.44. In the same manner, with respect to manageability and probability its idealized score respectively would rise up to 0.22 and drop down to 0.07.

## Description of results and discussion

Overall priorities of the alternatives, illustrated in Fig. 8, indicate that R12, Key customers’ reluctance in support of green initiatives or green products and services, R6, Failure to reach the quality expected of the building, R9, Scarcity of experts, Experienced and skilled labor, in the context of green construction, and R2, Unavailability of raw green materials and equipment, are the most important risk factors in descending order, with priority weights fluctuating between 0.21 and 0.1.

The above mentioned most important risk factors pivot on some key terms such as customers’ support, quality expected of the building which encompasses the notions of wellbeing during the construction phase up to the recycling period, professional workforce, and hard infrastructure like green material and equipment. Also, the latter is highly related to green process technology (please refer to R5) which is ranked the fifth most important key risk factor, with the importance weight of 0.6. However, these findings are also congruent with previous studies conducted in developing countries, in the realm of green building risks. In this regard, key risks identified in green building projects in china include quality and technical oriented risks such as failure in achieving the green building quality standard, health in built environment, and user behavior (Yang et al. 2016), lack of knowledge, professional workforce, technology, material and equipment, insufficient design with respect to local conditions, and inaccurate prediction of green market (Qin et al. 2016). In addition, Hwang et al. (2017) proposed key risk mitigation measures for green residential building projects in Singapore such as understanding the green building standards and upgrading skills and knowledge of new technologies and material.

Furthermore, sensitivity analysis indicates that priority weights of the most important risk factors identified are predicted to remain almost consistent when priority of the criteria fluctuates closer to the mid-point of each spectrum. However, some deviations were detected in the alternatives’ ranks and weights, given that greater changes would happen in priority weights of the criteria. Nevertheless, priority weights of the less important key risk factors were so close, leading to the conclusion that only a few number of these changes would have critical meanings. General indications of the sensitivity analysis are as the following.

The four most important risk factors, namely R12, R6, R9, and R2 do not show extreme sensitivity to rank changes regarding the weights of the criteria. But as the importance weight of the impact rises, their prominence increases, especially in R12 and R6. An increase in the importance of influence rate, in contrast, decreases the prominence of R12 and R6. The other two criteria, probability and manageability, would most likely just incur changes merely in the less important alternatives. In summary, the whole twelve alternatives examined are key risk factors, therefore all should be treated carefully, but some of these key elements are even more important. Although the results show menial sensitivity in case of even some dramatic change in circumstances, senior managers may have to consider some features more carefully, which are severity, likelihood, consequential situation, organizational resilience, and the influential interrelations.

Our developed method herein provides a comprehensive basis for risk management of green construction processes compared to other quite recent approaches: for example, Zhao et al. (2016) did not explicitly consider manageability and inter-risk influence triggers and considered only frequency, severity, and hence risk criticality, simply finding cost overruns were the key risk area. Our approach, using experts and combining their expertise in the extended risk framework, was able to assess and prioritize risk driving elements, that should prove more useful for project proponents than the more simplistic methods of the traditional ‘frequency severity’ approach.

Moreover, previous studies (Hwang et al. 2017; Qin et al. 2016) prioritized risk factors concerning their frequency of likelihood and magnitude of impact without taking into consideration the varying importance weigh of the criteria with respect to the alternatives. Another valuable privilege of the proposed model herein is the integration of fuzzy logic into ANP suing a discrete bell-shaped fuzzy function. While fuzzy arithmetic can allegedly digress the judgment matrices, but as we suggested herein using a discrete fuzzy function is supposed to safely maintain the initial judgments intact that otherwise can render invalid output (Saaty and Tran 2007). Other approaches such as resilience engineering (Rosa et al. 2017) are also not as comprehensive as those developed herein, either not using experts comprehensively, or not in any well-structured way processing comprehensive information and judgments on the four elements of frequency, severity, manageability, and influence. Further developments will include automating the algorithms, providing a widespread platform for use of this approach.

A further important discussion point is about the potential for generalization of the approach developed and illustrated herein. It is likely that some of the risk factors examined in this study (Table 6) will be common or at least very similar to those in other green construction project types. However, the priorities and value of these factors will likely be different, especially in different markets, and regulatory regimes. The methods will apply and be usefully comprehensive compared with traditional risk management approaches, even though the details of the priorities will not be identical to other green projects. In other types of construction, the risk elements are likely to be even more different from those herein, with less ‘green’ emphasis, yet the extended risk management approach will still be effective as a more comprehensive method than traditional approaches.

## Conclusion

### Managerial implications

This study demonstrates significant implications for those who manage green construction projects in particular and for projects of similar complexity in general terms. First, the traditional ‘frequency severity’ approach has been found to be lacking in consideration of two other important elements, namely manageability, and influence. Further, our illustrative case study showed how multiple experts’ judgments can be elicited and processed to rigorously determine priorities. At a base level, the application of this approach will alert managers to the acute risks and their importance in terms of not just the traditional magnitude factors (frequency and severity) but also the action and control factors (manageability and influence), including as to how risk factors are inter-reactive. This new approach gives professional managers a fully sound basis for prioritizing their interests and finite resources in risk management.

### Theoretical implications

The main purpose of this study was to conduct an integrated decision-making method to prioritize risk factors. In light of the importance of green residential megaprojects, its supply chain oriented risk factors were examined where twelve all-inclusive items were investigated through the ANP questionnaire. To bring more precision to the uncertain concept of risk management and judgmental decision making, the current paper proposed a novel and safe integration of ISM and Fuzzy logic to the ANP method. Furthermore, the row sensitivity analysis examined possible scenarios of the rank change in alternatives concerning change in the importance of the criteria. Some various conditions were examined and the most important alternatives and the stability of the results were discussed. The results indicate the instrumentality of the integrated methodology proposed. And although the resulting priorities are pertinent to the specified case under study, the practicality of the investigation in other cases is also supported, by providing the required material to reproduce the data analysis.

### Future research directions

Further examinations may consider examining the positive dependence and negative dependence of the risk factors on each other (Sarker et al. 2016). The row sensitivity analysis in this study examined the sensitivity of the alternatives regarding one criterion at a time, and further examinations may also consider multiple row sensitivity analysis. It is also suggested that the procedure proposed herein be administered in a future study in terms of a case company located in a developed country to compare likely similarities and or differences in the results.

### Limitations

This investigation has some limitations, for example, judgmental decision-making relies on the precision and the degree of expertise the expert panel has acquired. The ability to process the data is also necessary, as is the availability of panel experts. Also, the large number of pair-wise comparisons makes the questionnaire tedious for the expert panelists.

## Appendix

The RSA technique encompasses two ideas. The first is to force down entries of the row selected, and the second is to force the entries of that particular row up. Each element can be targeted. The variable parameter p (p-value), defined to monitor the changes. The resting p-value (p_0_) indicates the original standard values and equals 0.5. To push a row towards 0 means to reduce the importance of a particular corresponding node, while pushing a row towards 1 means to increase its importance. Simultaneously, the remaining rows must proportionately be changing inversely in the opposite direction. The following explanations are supposed to illuminate the calculations.

To scale down a row, entries of the selected row would be divided by any integer greater than 1. For example, if the entries of a row would be divided by x; for each entry (W_ij_) the remaining entries in that column should be multiplied by [1 – (W_ij_ ÷ x)] ÷ (1 – W_ij_). Doing so the column-wise summation of the entries will remain to be adding up to 1, which means the super-matrix is still stochastic (Saaty 2004). To scale up a selected row, entries would be added to N, where N = [(1 – W_ij_) ÷ x] and x > 1. Subsequently, the next step is to make the column-wise summations of the entries adding up to 1. This is done by multiplying corresponding entries in each column by [(1–N) ÷ (1 – W_ij_)].

To illuminate this procedure, the following tutorial illustrates how a particular row, of a given weighted matrix, can be scaled up and down. For instance, consider scaling up the middle row of the given 3X3 matrix below.

$$\left(\begin{array}{ccc}0.30& 0.10& 0.50\\ 0.20& 0.20& 0.40\\ 0.50& 0.70& 0.10\end{array}\right)$$

The first step is to multiple the selected row halfway towards 0 and the result is: (0.10 0.10 0.20).

The next step is to proportionately change the value of the entries in the remaining rows, and then normalize each column at a time. For the first column, the remaining entries used to add up to 0.8, and now they need to add up to 0.9, therefore, each entry should be multiplied by (0.9 ÷ 0.8). In the same manner, each entry in the second and the third column should be multiplied by (0.9 ÷ 0.8) and (0.8 ÷ 0.6) accordingly. The resulting matrix is shown below.

$$\left(\begin{array}{ccc}0.33& 0.11& 0.99\\ 0.10& 0.10& 0.20\\ 0.56& 0.78& 0.13\end{array}\right)$$

Now, to scale up the selected row, the middle row in our case, should be multiplied halfway towards 1 and the result is: (0.60 0.60 0.70). Once again, normalizing the remaining columns, for the first column, the remaining entries used to add up to 0.8 and now they need to add up to 0.4, therefore, each entry should be multiplied by (0.4 ÷ 0.8). In the same manner, each entry in the second and the third column should be multiplied by (0.4 ÷ 0.8) and (0.6 ÷ 0.3) accordingly. The resulting matrix is shown below.

$$\left(\begin{array}{ccc}0.15& 0.05& 0.25\\ 0.60& 0.60& 0.70\\ 0.25& 0.35& 0.05\end{array}\right)$$

The resulting influence analysis empowers the decision-makers to determine the most influential factors and the possibility of exploiting the following implications is being expected from the results.

First. Marginal influence: detecting the smallest changes in a selected criterion that incurs the most prominent changes in the alternatives?
Second. Rank influence: which of the criteria incurs a rank change in alternatives, the faster? In other words, which node incurs changes in rankings the first. Finding top influencers, where the least change in the node, brings about some changes in the alternatives.
Third. Node sensitivity: what are the rank changes of the alternatives at any given weight change regarding the criterion selected?
Fourth. Influence analysis: changing the weight of a particular node, in what manner ranks do change?
Fifth. Perspective analysis: what would the resulting ranking of the alternatives be, if the given parent node could receive the highest importance weight?

## Abbreviations

ANP: Analytic Network Process;
GSCM: Green supply chain management;
SCRM: Supply chain risk management;
MFs: Membership Functions
